# Histological evaluation of phospho‐epitopes within the core region of soluble and fibrillar tau

**DOI:** 10.1002/alz.092963

**Published:** 2025-01-09

**Authors:** Eric E Abrahamson, Anuradha Sehrawat, Xuemei Zeng, Tara K Lafferty, Lan Shao, Thomas K Karikari, Milos D Ikonomovic

**Affiliations:** ^1^ VA Pittsburgh Healthcare System, Pittsburgh, PA USA; ^2^ University of Pittsburgh School of Medicine, Pittsburgh, PA USA; ^3^ University of Pittsburgh, Pittsburgh, PA USA

## Abstract

**Background:**

Phosphorylated tau proteins accumulate in pathological aggregates which define neurodegenerative tauopathies, including Alzheimer’s disease (AD). Insight into the early stages of tau polymerization/aggregation, including early hyperphosphorylation events, is critical for identification of biomarkers of incipient disease as well as novel therapy targets.

**Method:**

We analyzed postmortem tissue sections of hippocampus from AD cases and middle frontal gyrus from non‐AD cases with mainly 4R tau isoforms (progressive supranuclear palsy, PSP; corticobasal degeneration, CBD; aging related tau astrogliopathy, ARTAG) or 3R tau (Pick’s disease, PiD). Immunohistochemical and multiple fluorescence methods were used to assess the labeling patterns of antibodies directed to phospo‐epitopes within soluble non‐fibrillar tau core (pSer262), both soluble and fibril core (pSer356), or outside of either core region (pSer202/pThr205, pThr231).

**Result:**

In sections of hippocampus from AD cases, pSer262 and pSer356 stained punctate/granular tau aggregates in pretangles while pSer202/pThr205 and pThr231 antibodies stained tau in pretangles as well as classic NFT, neuritic component of plaques, and neuropil threads. Both pSer262 and pSer356 signals co‐distributed with pSer202/pThr205 signal in pretangles but were rarely detected in pSer202/pThr205 immunoreactive classic NFT. In sections of middle frontal gyrus from PSP and CBD cases, tufted astrocytes and astrocytic plaques, respectively, were robustly labeled with the pSer202/pThr205 antibody but lacked significant immunoreactivity to pSer262 and pSer356 antibodies. Similarly, thorn shaped astrocytes in ARTAG cases were robustly labeled with the pSer202/pThr205 antibody but were weakly labeled with the pSer262 and pSer356 antibodies. Pick bodies were labeled robustly with the pSer202/pThr205 antibody, moderately with the pSer356 antibody, and weak and sparsely with the pSer262 antibody.

**Conclusion:**

Antibodies directed against phosphorylated epitopes within core region of soluble and fibrillar tau distinguish hippocampal pretangles in AD and appear to have less affinity for tau pathology forms in non‐AD tauopathies. Thus, they may be novel biomarkers for detecting early stages of neurofibrillary pathology development in AD.

